# Readiness of Advance Care Planning Among Patients With Cardiovascular Disease

**DOI:** 10.3389/fcvm.2022.838240

**Published:** 2022-06-02

**Authors:** Noriko Fukue, Emiko Naito, Masayasu Kimura, Kaoru Ono, Shinichi Sato, Akira Takaki, Yasuhiro Ikeda

**Affiliations:** ^1^Department of Cardiology, Tokuyama Medical Association Hospital, Shunan, Japan; ^2^Department of Cardiology, Yamaguchi Prefectural Grand Medical Center, Hofu, Japan; ^3^Nursing Department, Tokuyama Medical Association Hospital, Shunan, Japan

**Keywords:** advance care planning, readiness, cardiovascular disease, predictive factors, end-of-life

## Abstract

**Background:**

Advance care planning (ACP) is a widely advocated strategy to improve outcomes at end-of-life care for patients suffering from heart failure (HF). However, finding the right time to start ACP is challenging for healthcare providers because it is often a sensitive issue for patients with HF and their families. We interviewed patients with cardiovascular diseases regarding ACP readiness and investigated the relationship between the ACP desire and multiple clinical prognostic parameters.

**Method:**

Eighty-one patients (average age 81.8 ± 10.3 years old, 42 men, 62 cases of HF) who introduced cardiac rehabilitation were inquired about previous ACP experience, a desire for ACP, understanding of their cardiovascular diseases, and lifestyle-associated questionnaires. Multiple logistic regression analyses were employed to identify the clinical parameters associated with ACP desire. Patients who desired ACP were also asked about their preferences for medical care at the end-of-life.

**Results:**

Nine patients (11.1%) had previous experience with ACP, and 28 (34.6%) preferred to implement ACP. Patients who did not want to implement ACP were 54.3%. Patients with HF showed a higher acceptance rate of ACP (odds ratio [OR] 5.56, *p* = 0.015). Interestingly, patients harboring skeletal muscle frailty showed lower ACP acceptance, while patients with non-frailty rather positively wanted to implement ACP. Two types of prognosis evaluation scales, such as the Enhanced Feedback for Effective Cardiac Treatment (EFFECT) risk score and the Japanese Version of Supportive and Palliative Care Indicators Tool (SPICT-JP), identified 31 patients (38.3%) needing ACP; however, 19 (61.3%) did not want ACP. The wish not to attempt resuscitation and life-prolonging treatment at the end-of-life reached approximately 70% among patients who requested ACP.

**Conclusions:**

Although patients with HF tended to be ready for implementing ACP, the presence of skeletal muscle frailty was negatively associated with ACP preference. Indeed, patients who should be considered ACP were not carried out and did not desire it. Earlier introduction of ACP into patients before having skeletal muscle frailty may be considered.

## Introduction

The number of elderly patients with heart failure (HF) repeatedly admitted to hospitals due to acute exacerbations increases with the aging society. In many cases, symptoms during acute exacerbations improve quickly with treatment, so it is known that both patients and healthcare providers have dissociated perceptions of prognosis from reality. It is important to conduct advance care planning (ACP) to prepare for future conditions, such as the terminal stage. The goal is for the patient to lead a satisfying life at the end-of-life. ACP has been reported to improve clinical outcomes (1–5), not to increase anxiety, depression, and hopelessness in patients (6–11), reduce distress in surrogate decision-makers (5, 12), and reduce costs (13). Lack of proper communication about end-of-life preferences leads to lower quality of life, patient anxiety and family distress, the prolonged dying process, unwanted hospitalizations, distrust of medical care, physician burnout, and higher costs (14). However, it is often difficult for patients to face death themselves at the terminal stage, and it is difficult for medical professionals to broach the topic. ACP should be performed when the patient's readiness is in order (15) but it is not easy to confirm this condition.

Therefore, we decided to survey cardiac rehabilitation patients, i.e., relatively healthy patients with cardiovascular disease, about their experience and desire for ACP implementation.

## Methods

Consecutive patients who were introduced to first cardiac rehabilitation at the Tokuyama Medical Association Hospital between July 2019 and August 2021 were assessed for study inclusion. The questionnaire was used to survey patients before cardiac rehabilitation (Table 1). Briefly, we asked patients about their previous ACP experience, their desire for ACP, their diagnosis, and lifestyle-associated questionnaires. Patients who had previous experience with ACP or expressed a desire to implement ACP were categorized as the ACP preferred group. Patients who did not want to receive ACP were regarded as the ACP un-preferred group in the following text and tables.

**Table 1:** Questionnaire at the time of initiation of cardiac rehabilitation.

**Q1. For what disease have you been advised to undergo rehabilitation this time? If you have heart failure, what has been explained by your doctor as the cause of your heart failure? Please describe to the extent you can understand**.
**Q2. What symptoms are you currently experiencing?** **Shortness of breath on exertion/chest pain on exertion/leg pain on exertion/palpitations/swelling/Other**
**Q3. Do you have an exercise habit in your daily life?** **No/Yes Please specify the type and frequency of your exercise**.
**Q4. Please check all that apply for your current residence.** **Home / Institution / Other**
**Q5. Please check all that apply to your family members who live with you.** **Husband / Wife / Son / Daughter / Grandson / Other**
**Q6. In case of an emergency, if you are unable to confirm your intentions, who can you ask to make decisions on your behalf?** Name: **Relationship: Contact information (phone number)**
**Q7. Are you currently working?** **Yes/ No/ On leave and planning to return to work**
**Q8. Are you currently a cigarette smoker?** **Yes/No**
**Q9. If you are a current or former smoker, please tell us how many cigarettes you smoke per day and how long you have smoked.** **( ) cigarettes/day, ( ) years**
**Q10.If you are a drinker, what is your average amount of alcohol consumed per day and days per week?** **( )/day, ( )/week**
**Q11. Do you experience choking when you drink or eat?** **Often/ Sometimes/ Almost never**
**Q12. Do you have a heart failure certificate?** **Yes/No**
**Q13. Do you have a pacemaker or other device implanted in your body?** **Yes/No**
**Q14. If there is a patient class where you can learn about cardiovascular diseases and what you should do in your daily life, would you like to attend?** **Yes/No**
**Q15. Do you know what benefits can be expected from cardiovascular rehabilitation?** **Yes/No**
**Q16. Have you talked with your doctors or other medical professionals about what kind of treatment you want or do not want to receive, where you want to spend your time, etc. in the end-stage of your illness?** **With a doctor / With a medical professional other than a doctor / None**
**Q17. Would you like to discuss the above?** **Yes/No**

Clinical variables were obtained from the medical records. These include the following variables; i.e., the existence of HF, New York Heart Association (NYHA) classification, left ventricular ejection fraction (LVEF), Controlling Nutrition Status (CONUT) score, Geriatric Nutritional Risk Index (GNRI), body mass index (BMI), Functional Independence Measure (FIM), and Short Physical Performance Battery (SPPB) collected within a week following the initiation of cardiac rehabilitation.

The CONUT score is calculated from serum albumin, total cholesterol concentrations, and total lymphocyte count, and evaluated nutrition status as follows: 0–1 point as normal, 2–4 points as mild, 5–8 points as moderate, and >8 points as severe malnutrition (16, 17). The GNRI is a nutritional risk index published by Bouillanne et al. in 2005 and is calculated by the formula of [1.489 × serum albumin (g/L) + 41.7 × body weight (kg)/ideal weight (kg)] (18). Its prognostic value has been evaluated in elderly patients, hemodialysis patients, and HF patients. From GNRI values, they defined four grades of nutrition-related risk: major risk (GNRI: <82), moderate risk (GNRI: 82 to <92), low risk (GNRI: 92 to <98), and no risk (GNRI: >98) (18). The FIM is an activity of daily life assessment method that includes motor and cognitive items and is scored on a scale of 18–126 (19). The SPPB is an index for evaluating lower limb function in the elderly and is based on a 4-point scale for balance, gait, and standing (20). Eight or fewer points were regarded as frail (19). Personal health records and questionnaires confirmed the coexistence of cancer and chronic obstructive pulmonary disease (COPD), history of aspiration pneumonia, and cerebrovascular disease. The coexistence of dementia was identified as less than 21 points of Mini-Mental State Examination (MMSE) or administration of oral dementia drug.

The Enhanced Feedback for Effective Cardiac Treatment (EFFECT) risk score and the Supportive and Palliative Care Indicators Tool Japanese Version (SPICT-JP) were used as prognostic scales (21–23). The EFFECT risk score predicts 30-day and 1-year mortality by using the following factors: age (year), respiratory rate (breaths/min), systolic blood pressure (mmHg), blood urea nitrogen (mg/dl), presence of sodium concentration <136 mEq/L, cerebrovascular disease, dementia, COPD, hepatic cirrhosis, cancer, and the value of hemoglobin <10.0 g/dl. Patients with very low-risk scores ( ≤ 60) had a mortality rate of 0.4% at 30 days and 7.8% at 1 year. Patients with very high-risk scores (>150) had a mortality rate of 59.0% at 30 days and 78.8% at 1 year (21).

**Figure 1:**
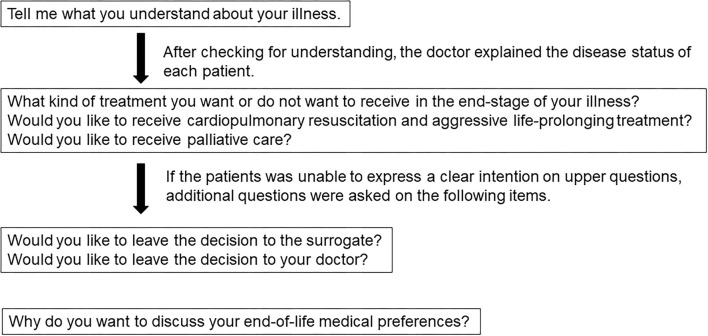
Interview about the preferences for the end-of-life care.

The SPICT consists of a combination of general clinical indicators (e.g., poor performance status, unplanned hospital admissions, or persistent symptoms despite optimal treatment of the underlying condition) relevant to patients with any advanced illness and disease-specific indicators for common advanced conditions (e.g., cancer, dementia, and cardiac, pulmonary, or renal disease) (22). It has been reported that four multidisciplinary teams identified 130 patients with advanced kidney, liver, cardiac, or lung disease following an unplanned hospital admission. Hospital clinicians used the SPICT to identify patients at risk of deteriorating and dying. Patients who died had significantly more frequent unplanned admissions, persistent symptoms, and increased care needs. By 12 months, 62 (48%) of the identified patients had died; 69% of them died in hospital, having spent 22% of their last 6 months there (22). One report shows that the SPICT identified patients with palliative care needs better than the surprise questions commonly used in clinical practice. The sensitivity of the surprise question became 69%, of the SPICT 81% regarding predicting 1-year mortality (24). The SPICT-JP is a Japanese version of the SPICT tool (23). The SPICT-JP positive is defined as the presence of two or more of the general indicators of deteriorating health or one or more of the clinical indicators of an advanced state of each disease. Patients who requested ACP in the questionnaire were asked about their preferences for end-of-life care to the extent possible. The dialogue content was prepared regarding previous studies (25, 26). The physicians conducted the interviews following the procedure shown in Figure 1.

The experience with ACP was defined as those who answered “With a doctor” or “With a medical professional other than a doctor” to “Q16. Have you talked with your doctors or other medical professionals about what kind of treatment you want or do not want to receive, where you want to spend your time, etc., in the end-stage of your illness?” of the questionnaire. The desire of ACP was defined as those who answered “Yes” to “Q17. Would you like to discuss the above?” of the questionnaires. The end-of-life was defined as a 1-year mortality rate >50%, according to the EFFECT risk score or positive SPICT-JP in this study.

Patients were informed of the publication of the survey results, obtained with the individual's consent. This protocol received approval from the Ethics Committee of Tokuyama Medical Association Hospital (*approval number*: 12), and it conformed to the Declaration of Helsinki provisions.

### Statistical Analysis

The patients' backgrounds were compared between the ACP preferred patients and un-preferred patients using the Fisher's exact test for categorical variables, unpaired *t-*test for continuous normative data, and Mann-Whitney U test for non-normative continuous data. Univariate and multiple logistic regression analyses were employed to analyze the association between ACP preference and clinical prognostic parameters. Independent variables for multiple logistic analysis were selected from three predictive factors with *p* <0.15 using univariate analysis, HF, CONUT score as the nutritional status, and SPPB as the degree of frailty. The probabilities of higher EFFECT risk score and SPICT-JP positive group were compared between the ACP preferred group and the unpreferred group using Fisher's extract method.

All statistical analyses were performed with EZR (27). Briefly, it is a modified version of R commander designed to add statistical functions frequently used in biostatistics, and results with a value of *p* <0.05 were considered statistically significant.

## Results

Of the 100 patients initially introduced to cardiac rehabilitation at the Tokuyama Medical Association Hospital, 81 answered the questionnaire. The patient characteristics are shown in Table 2. The mean age was 81.8 years, 58 (71.6%) patients were hospitalized, 62 (76.5%) patients had HF with stage C, or more advanced stage, 30 (37.0%) patients had dementia, the mean GNRI was 93.6, and the mean SPPB was 6.8. Nine patients (11.1%) had previous experience with ACP, and 28 (34.6%) patients did not perform but were willing to implement ACP. Comparing patients in the ACP preferred (*n* = 37) and unpreferred groups (*n* = 44), there were significant statistical differences in the CONUT score, SPPB, and FIM groups. Multivariate analysis showed further significant differences in HF (odds ratio [OR] 5.56, *p* = 0.015) and SPPB (OR 1.25, *p* = 0.006; Table 3). These data indicate that patients with stage C or advanced HF showed a higher acceptance rate of ACP. In addition, patients harboring lower body skeletal muscle frailty (low SPPB score) showed a lower preference for ACP. In contrast, patients with non-frailty (SPPB score >8) tended to want to implement ACP.

**Table 2:** Patient characteristics.

	**Total (*n =* 81)**
Age(years), mean ± SD	81.8 ± 10.3
Gender	
Male, *n* (%)	42 (51.9)
Female, *n* (%)	39 (48.1)
Inpatients, *n* (%)	58 (71.6)
Outpatients, *n* (%)	23 (28.4)
Living situation	
Cohabitation with family, *n* (%)	45 (55.6)
Separation, *n* (%)	36 (44.4)
Cardiovascular disease	
Heart failure, *n* (%)	62 (76.5)
IHD, *n* (%)	31 (38.3)
Atrial fibrillation, *n* (%)	39 (48.1)
After open heart surgery, *n* (%)	6 (7.4)
Aortic disease, *n* (%)	4 (4.9)
PAD, *n* (%)	23 (28.4)
HT, *n* (%)	57 (70.4)
Non-cardiovascular disease	
CKD	60 (74.1)
Dyslipidemia, *n* (%)	34 (42.0)
DM, *n* (%)	26 (32.1)
Cancer, *n* (%)	5 (6.2)
Dementia, *n* (%)	30 (37.0)
COPD, *n* (%)	7 (8.6)
History of aspiration pneumonia, *n* (%)	3 (3.7)
History of cerebral vascular disease, *n* (%)	15 (18.5)
Evaluation items	
Understanding of the disease	31 (38.3)
NYHA, median (IQR)	3 (2,3)
BMI, mean ± SD	22.1 ± 3.7
LVEF, mean ± SD	51.4 ±15.6
CONUT score, median (IQR)	4 (2,5)
GNRI, mean ± SD	93.6 ± 10.8
SPPB, mean ± SD	6.8 ± 3.8
FIM, median (IQR)	99 (79, 119)

*Values were shown as mean ± standard deviation (SD), median (interquartile range (IQR): 25th to 75th percentiles), n (%). IHD, ischemic heart disease; PAD, peripheral artery disease; HT, hypertension; CKD, chronic renal disease; DM, diabetes mellitus; COPD, chronic obstructive pulmonary disease; NYHA, New York Heart Association classification; BMI, body mass index; LVEF, left ventricular ejection fraction; CONUT score, controlling nutritional status; GNRI, geriatric nutrition risk index; SPPB, Short Physical Performance Battery; FIM, Functional Independence Measure*.

**Table 3:** Univariate and multivariate analyses to predict preference of ACP.

	**Univariate analysis**			**Multivariate analysis**		
	**ACP preferred**	**ACP un-preferred**	***P* value**	**OR**	**95%CI**	***P* value**
	(*n =* 37)	(*n =* 44)				
Age (years), mean ± SD	81.4± 12.0	82.0 ± 8.8	0.78			
Male, *n* (%)	22 (59.5)	20 (45.5)	0.27			
Outpatients, *n* (%)	14 (37.8)	9 (20.5)	0.14			
Heart failure, *n* (%)	32 (86.5)	30 (69.8)	0.11	5.56	1.39–22.20	0.015*
Cancer, *n* (%)	2 (5.4)	3 (6.8)	1			
Dementia, *n* (%)	12 (32.4)	18 (40.9)	0.70			
COPD, *n* (%)	2 (11.4)	5 (11.4)	0.45			
History of aspiration pneumonia, *n* (%)	1 (2.7)	2 (4.5)	1			
History of cerebral vascular disease, *n* (%)	7 (18.9)	8 (18.2)	1			
Separation, *n* (%)	16 (43.2)	20 (45.5)	1			
Understanding of the disease, *n* (%)	15 (40.5)	16 (36.4)	0.82			
NYHA, median (IQR)	2 (2,3)	3 (2,3)	0.58			
BMI, mean ± SD	22.1 ± 3.1	22.1 ± 4.2	0.91			
LVEF, mean ± SD	47.7 ± 17.4	54.4 ± 13.5	0.06			
CONUT score, median (IQR)	3 (2,5)	4 (3,6)	0.005*	0.82	0.65–1.03	0.087
GNRI, mean ± SD	95.9 ± 10.7	91.6 ± 10.8	0.07			
SPPB, mean ± SD	8.2± 3.3	5.7 ± 3.8	0.002*	1.25	1.07–1.48	0.006*
FIM, median (IQR)	109(92, 124)	95 (70, 114)	0.009*			

* *Indicates p <0.05. In univariate analysis, age, BMI, LVEF, GNRI, and SPPB were compared using an unpaired t-test for continuous normative data. NYHA, CONUT score, and FIM were compared using the Man-Whitney U test for non-normative continuous variables, and others were compared using Fisher's exact test for non-continuous variables. Predictors of preference of ACP were identified by logistic regression analysis. Independent variables were selected from predictive factors with p <0.15 using univariate analysis. OR, odds ratio; CI, confidence interval; COPD, chronic obstructive pulmonary disease; NYHA, New York Heart Association classification; BMI, body mass index; LVEF, left ventricular ejection fraction; CONUT score, controlling nutritional status; GNRI, geriatric nutrition risk index; SPPB, Short Physical Performance Battery; FIM, Functional Independence Measure*.

Twenty-nine patients (35.8%) were predicted to have a mortality of ≥50% within 1 year by the EFFECT risk score (poor prognosis group); 32.4 and 38.6% of patients in the ACP preferred and unpreferred groups, respectively (no significant difference between the two groups). In contrast, 13 out of 81 patients (16.0%) were judged to have a poor (positive) prognosis using the SPICT-JP tool. The poor prognosis group was less frequent in the ACP preferred group and more frequent in the ACP unpreferred group (*p* = 0.03). These results indicate that patients who were judged as SPICT-JP positive tended not to prefer ACP (Table 4).

**Table 4:** Relationship between estimated prognosis and advance care planning (ACP) preference.

	**Total**	**ACP** **preferred**	**ACP** **un-preferred**	***P* value**
	**(*n =* 81)**	**(*n =* 37)**	**(*n =* 44)**	
1.1-year mortality rate >50% due to the EFFECT risk score, *n* (%)	29 (35.8)	12 (32.4)	17 (38.6)	0.64
2. SPICT-JP positive, *n* (%)	13 (16.0)	2 (5.4)	11 (25.0)	0.03*
1. and/or 2. positive, *n* (%)	31 (38.3)	12 (32.4)	19 (43.2)	0.37

* *Indicates p <0.05. The difference in the experience or desire for ACP between the poor prognosis group and the not poor prognosis group identified by the Enhanced Feedback for Effective Cardiac Treatment (EFFECT) risk score and Japanese Version of Supportive and Palliative Care Indicators Tool (SPICT-JP) was compared using Fisher's exact test for non-continuous variables*.

At the time of this survey, 31 patients had expressed a preference for ACP, and doctors conducted ACP dialogues with 24 of these patients (2 with previous ACP experience and 22 with no previous ACP experience). Table 5 examines the 24 patients' wishes regarding their medical care. Do-not-attempt-resuscitation (DNAR) accounted for 70.8% of the patients' end-of-life medical care. There was no significant difference in this medical preference between patients who were judged to be terminal by the EFFECT risk score or SPICT-JP and those who were not judged to be terminal by the EFFECT risk score or SPICT-JP. The reasons for requesting ACP were as follows: old age 10 (41.7%), no specific reason 7 (29.7%), aversion to life-prolonging treatment 5 (20.8%), and living alone 3 (12.5%).

**Table 5:** Medical Preferences among patients who requested advance care planning (ACP).

	**Total**	**End-of-life**	**Not** **end-of-life**	***P* value**
	**(*n =* 24)**	**(*n =* 9)**	**(*n =* 15)**	
Aggressive life-prolonging treatment, *n* (%)	0 (0)	0 (0)	0 (0)	
Do not attempt resuscitation and life-prolonging treatment, *n* (%)	17 (70.8)	6 (66.7)	11 (73.3)	1.00
Palliative care, *n* (%)	6 (25.0)	3 (33.3)	3 (20.0)	0.63
Leave the decision to the surrogate, *n* (%)	3 (12.5)	2 (22.2)	1 (6.7)	0.53
Leave the decision to their doctor, *n* (%)	6 (25.0)	3 (33.3)	3 (20.0)	0.63

*The differences in medical preferences between the end-of-life and not end-of-life groups were compared using Fisher's exact test for categorical variables. The “end-of-life” group was identified by a 1-year mortality rate >50% according to EFFECT risk score or Japanese Version of Supportive and Palliative Care Indicators Tool (SPICT-JP) positive*.

## Discussion

The surprise question has been widely used to determine the timing of ACP (28). However, the prognosis of HF is difficult to predict, and advance directives are rarely performed even when the patient is judged to be terminal stage by cardiologists (29). Patients with HF are more optimistic than clinicians in estimating life expectancy (30). Barriers to implementing ACP for healthcare providers include not understanding how to proceed with discussion, a concern that it may cause psychological distress to the patient, the desire to avoid the topic of end-of-life, the desire to avoid dealing with death anxiety, time constraints, difficulty in predicting prognosis, and lack of understanding of how to apply the ACP process to care (14). Factors on the patient's side include anxiety, denial, and a desire not to bother the family (14). Patients facing life-threatening situations tend to avoid discussing end-of-life issues. Other reports have shown that only 47% of patients with symptomatic HF could complete an advance directive, despite appropriate approaches (31). This study also showed that patients who were identified as having a poor prognosis by HF patients with physical frailty and SPICT-JP did not desire ACP. It was considered difficult to implement ACP for patients in the end-stage of HF, although palliative care is recommended. Investigations in previous studies have been dedicated to topics related to ACP (25, 26, 32, 33). The questionnaire in this study, which asked about lifestyle and social factors, showed that patients involved in ACP discussions were less willing to do so voluntarily. These patients may need to be encouraged and informed about ACP by the medical profession. A study of ACP readiness in patients with advanced lung and colorectal cancer reported that patients did not have to be ready for all ACP topics. They were able to participate in an ACP conversation (26).

On the other hand, such a questionnaire seems to be a good way to pick up ACP wishes in a group of patients who do not yet have a poor prognosis. Patients, who had HF, maintained muscle strength, and had been still far from a poor prognosis, were more willing to perform ACP. Although ACP for patients in situations far from death is considered impractical, initiating dialogue to explore the patient's values at the first ACP can be expected to lower the hurdle of ACP for both the patient and the medical profession. There was no difference in the preference for end-of-life care between the good prognosis group and the poor prognosis group. Moreover, about 70% of the patients in both groups expressed their intention to DNAR. Patients who reported to have an end-of-life conversation were more likely to report peacefulness and desire and received less-invasive care (2).

In a large study of patients older than 60 years, of those who required decisions, 70% did not have decision-making capacity, leaving decisions to surrogates or to previous advance directives (5). Japan has a universal healthcare system. Our healthcare system is oriented toward providing life-sustaining treatment and tends to provide intensive medical care for the elderly. In Japan, there is a tradition of abhorrence of death and a cultural background that makes it difficult to mention death. The HF pandemic is a major problem in Japan's aging society. It has become a vexing issue for medical professionals regarding how far they should go in providing treatment to frail elderly patients (34). In recent years, the ACP has been promoted as a national policy, and in 2018, the ACP was nicknamed the “Life Conference.” The number of people interested in ACP increases due to these educational activities, but medical staff cannot conduct ACP. This study suggests that ACP should be administered gradually to patients with the cardiovascular diseases earlier than we had assumed.

## Limitations

This study has several limitations. First, this research was a single-center study and a small sample size. Further studies with larger samples and multicenter enrollment need to be considered. Second, this study was exclusively Japanese and did not include other races, such as African American, White, Pacific, or others.

## Conclusions

Nearly half of patients with cardiovascular diseases introducing cardiac rehabilitation expressed a preference for ACP. Although patients with HF tended to be ready for implementing ACP, skeletal muscle frailty was negatively associated with ACP acceptance. Patients who should be considered ACP were not carried out and did not desire it. Earlier introduction of ACP into patients before having frailty may be considered.

## Data Availability Statement

The raw data supporting the conclusions of this article will be made available by the authors, without undue reservation.

## Ethics Statement

The studies involving human participants were reviewed and approved by the Ethics Committee of Tokuyama Association Hospital (Approval Number: 12). The patients/participants provided their written informed consent to participate in this study.

## Author Contributions

NF was the primary investigator for this study, collected data, and the overall writing of the project. YI supervised the writing of this paper, reviewed all documents, and helped to analyze the data, figures, and tables. EN administered the questionnaire. MK, KO, SS, and AT reviewed the manuscript and offered insights based on their experiences. All authors gave final approval, agreed to be accountable for all aspects of the work, and ensuring integrity and accuracy.

## Conflict of Interest

The authors declare that the research was conducted in the absence of any commercial or financial relationships that could be construed as a potential conflict of interest.

## Publisher's Note

All claims expressed in this article are solely those of the authors and do not necessarily represent those of their affiliated organizations, or those of the publisher, the editors and the reviewers. Any product that may be evaluated in this article, or claim that may be made by its manufacturer, is not guaranteed or endorsed by the publisher.
